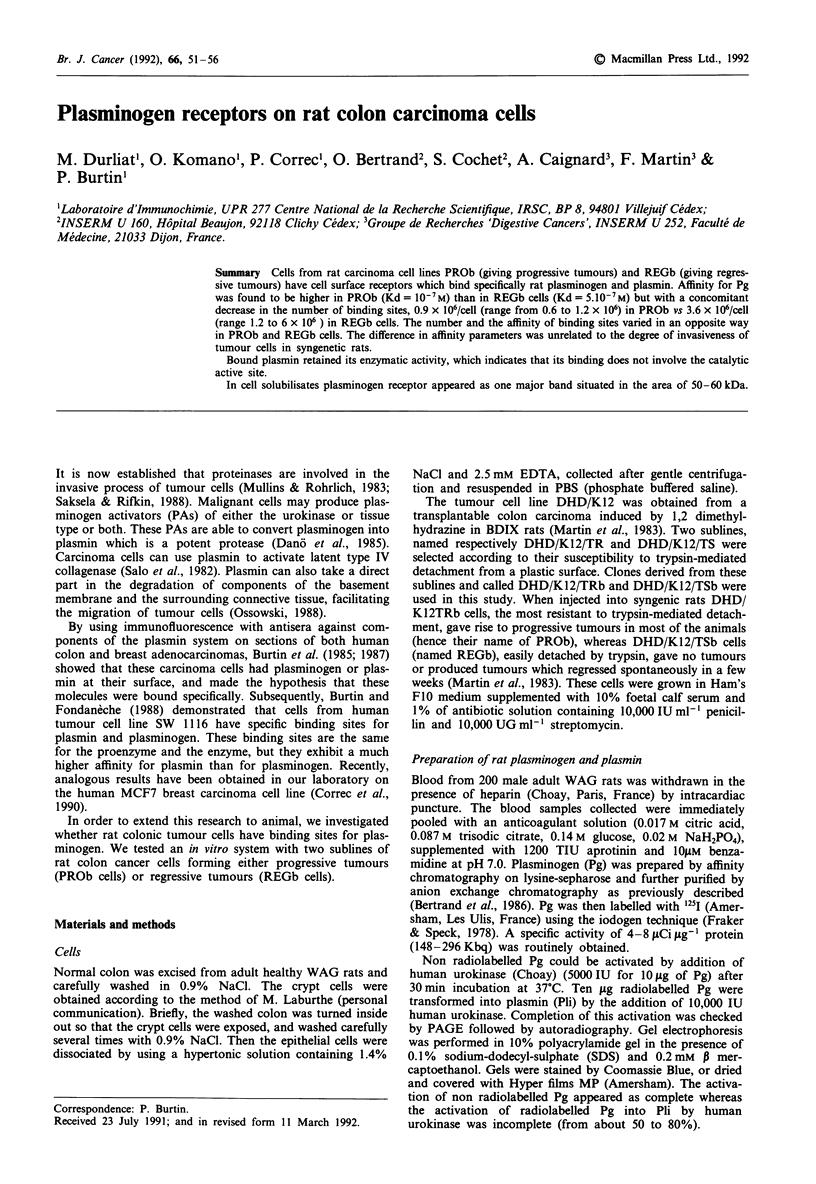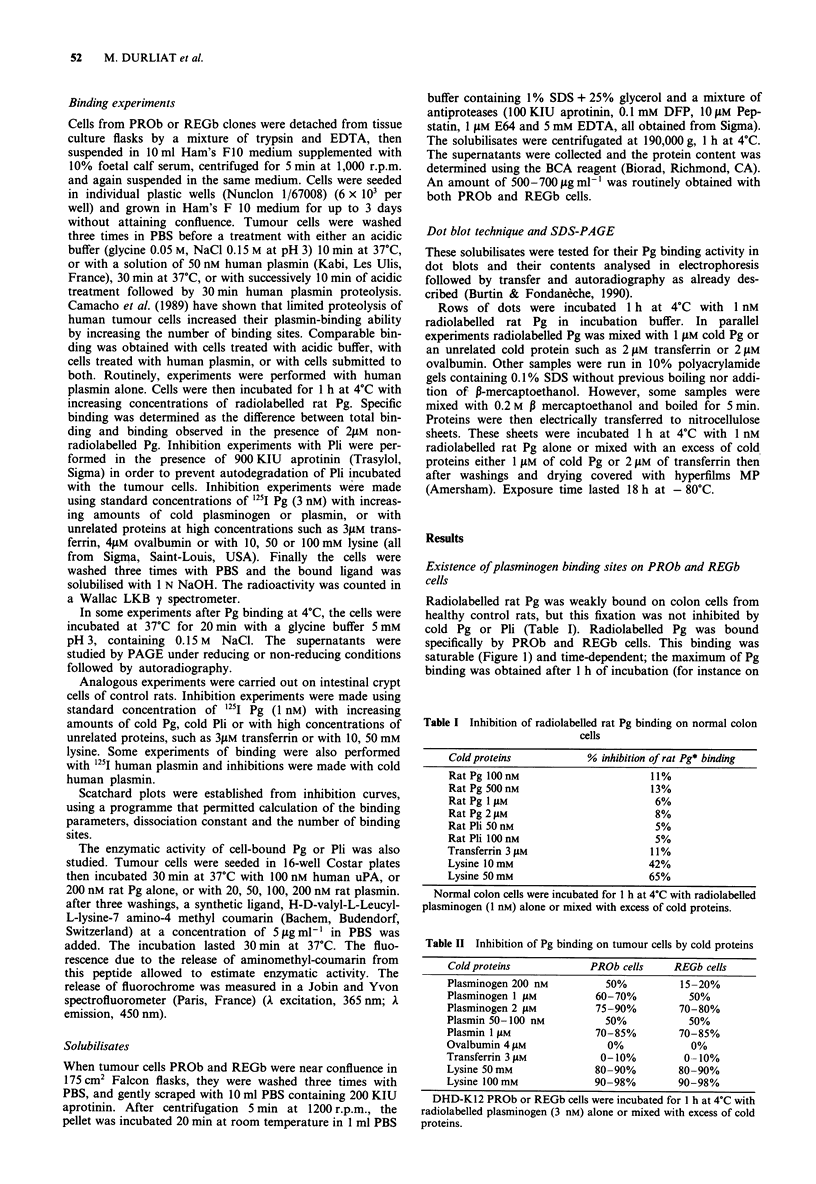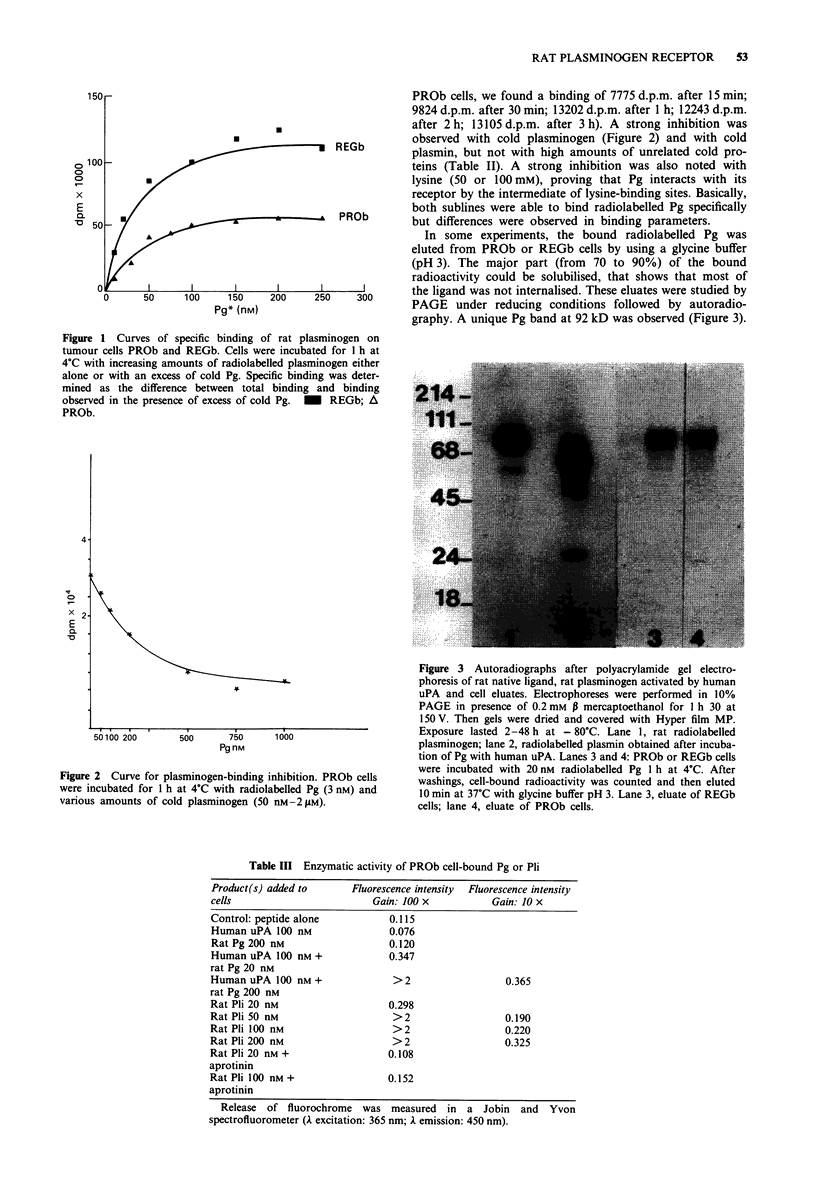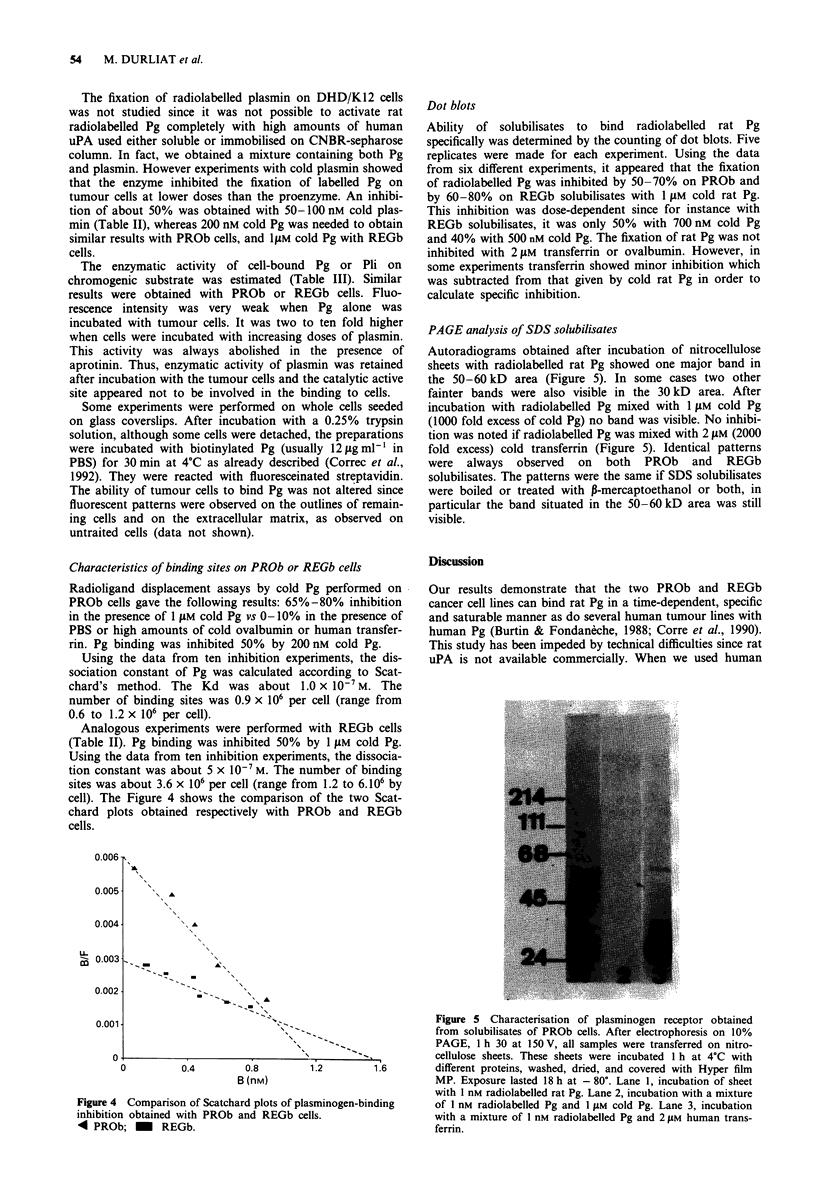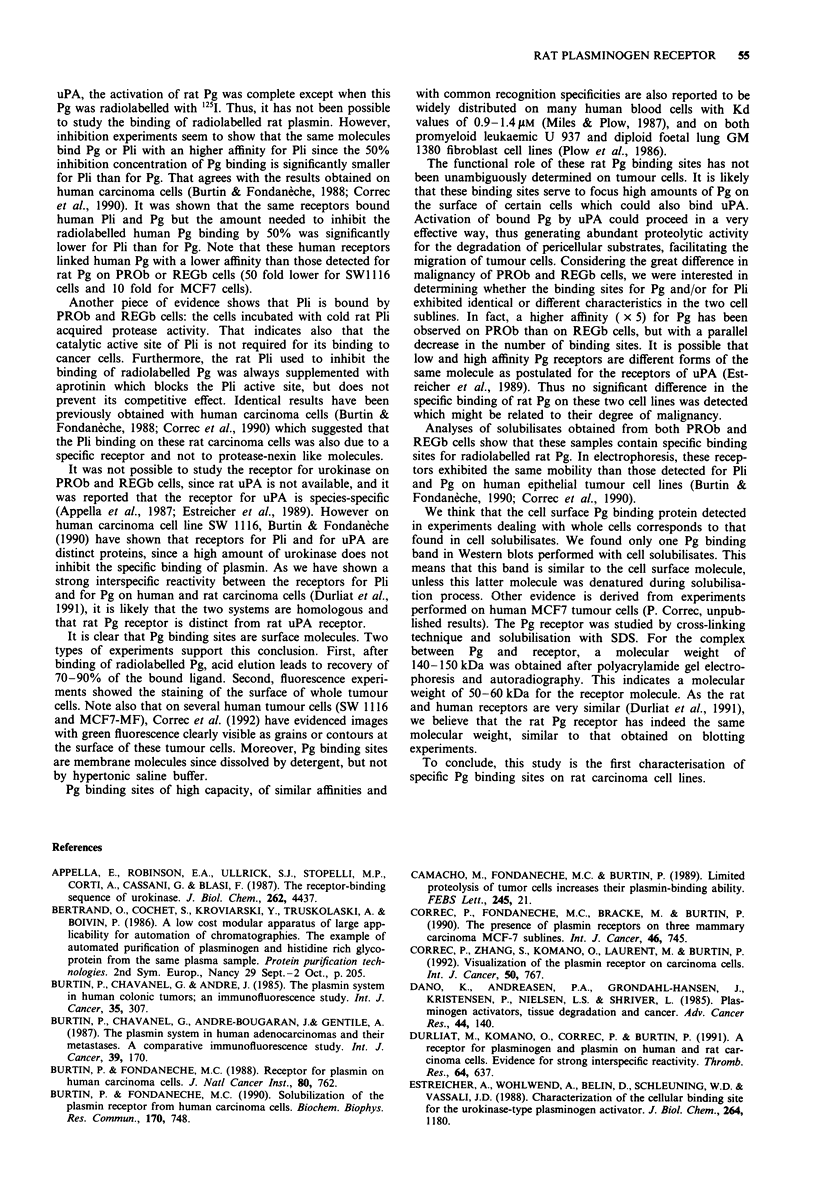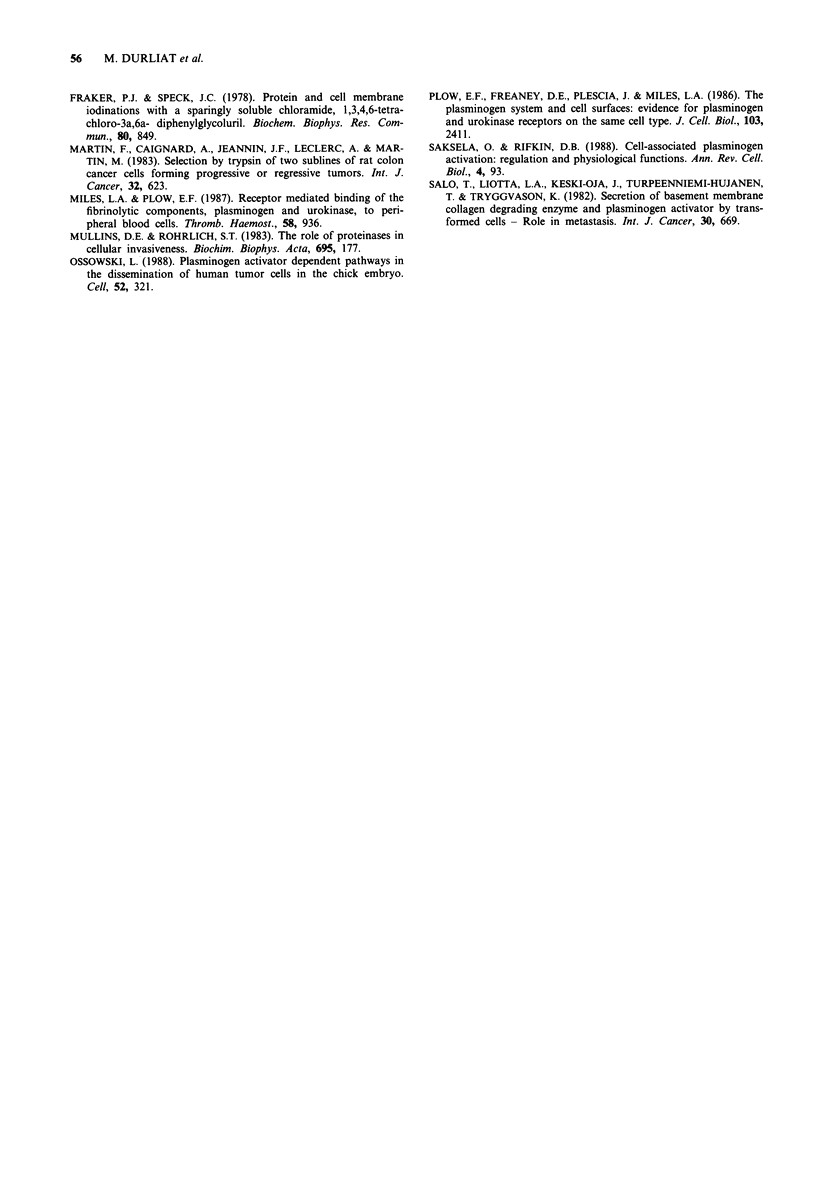# Plasminogen receptors on rat colon carcinoma cells.

**DOI:** 10.1038/bjc.1992.215

**Published:** 1992-07

**Authors:** M. Durliat, O. Komano, P. Correc, O. Bertrand, S. Cochet, A. Caignard, F. Martin, P. Burtin

**Affiliations:** Laboratorie d'Immunochimie, UPR 277 Centre National de la Recherche Scientifique, IRSC, Villejuif, France.

## Abstract

**Images:**


*Br. J. Cancer* (1992), **66**, 51–56 © Macmillan Press Ltd., 1992

### Plasminogen receptors on rat colon carcinoma cells

M. Durliat[1], O. Komano[1], P. Correc[1], O. Bertrand[2], S. Cochet[2], A. Caignard[3], F. Martin[3] & P. Burtin[1]

[1]*Laboratoire d'Immunochimie, UPR 277 Centre National de la Recherche Scientifique, IRSC, BP 8, 94801 Villejuif Cédex;* [2]*INSERM U 160, Hôpital Beaujon, 92118 Clichy Cédex;* [3]*Groupe de Recherches 'Digestive Cancers', INSERM U 252, Faculté de Médecine, 21033 Dijon, France.*

**Summary** Cells from rat carcinoma cell lines PROb (giving progressive tumours) and REGb (giving regressive tumours) have cell surface receptors which bind specifically rat plasminogen and plasmin. Affinity for Pg was found to be higher in PROb (Kd = $10^{-7}$ M) than in REGb cells (Kd = $5.10^{-7}$ M) but with a concomitant decrease in the number of binding sites, $0.9 \times 10^6$/cell (range from 0.6 to $1.2 \times 10^6$) in PROb *vs* $3.6 \times 10^6$/cell (range 1.2 to $6 \times 10^6$ ) in REGb cells. The number and the affinity of binding sites varied in an opposite way in PROb and REGb cells. The difference in affinity parameters was unrelated to the degree of invasiveness of tumour cells in syngenetic rats.

Bound plasmin retained its enzymatic activity, which indicates that its binding does not involve the catalytic active site.

In cell solubilisates plasminogen receptor appeared as one major band situated in the area of 50–60 kDa.

It is now established that proteinases are involved in the invasive process of tumour cells (Mullins & Rohrlich, 1983; Saksela & Rifkin, 1988). Malignant cells may produce plasminogen activators (PAs) of either the urokinase or tissue type or both. These PAs are able to convert plasminogen into plasmin which is a potent protease (Danö *et al.*, 1985). Carcinoma cells can use plasmin to activate latent type IV collagenase (Salo *et al.*, 1982). Plasmin can also take a direct part in the degradation of components of the basement membrane and the surrounding connective tissue, facilitating the migration of tumour cells (Ossowski, 1988).

By using immunofluorescence with antisera against components of the plasmin system on sections of both human colon and breast adenocarcinomas, Burtin *et al.* (1985; 1987) showed that these carcinoma cells had plasminogen or plasmin at their surface, and made the hypothesis that these molecules were bound specifically. Subsequently, Burtin and Fondanèche (1988) demonstrated that cells from human tumour cell line SW 1116 have specific binding sites for plasmin and plasminogen. These binding sites are the same for the proenzyme and the enzyme, but they exhibit a much higher affinity for plasmin than for plasminogen. Recently, analogous results have been obtained in our laboratory on the human MCF7 breast carcinoma cell line (Correc *et al.*, 1990).

In order to extend this research to animal, we investigated whether rat colonic tumour cells have binding sites for plasminogen. We tested an *in vitro* system with two sublines of rat colon cancer cells forming either progressive tumours (PROb cells) or regressive tumours (REGb cells).

### Materials and methods

### *Cells*

Normal colon was excised from adult healthy WAG rats and carefully washed in 0.9% NaCl. The crypt cells were obtained according to the method of M. Laburthe (personal communication). Briefly, the washed colon was turned inside out so that the crypt cells were exposed, and washed carefully several times with 0.9% NaCl. Then the epithelial cells were dissociated by using a hypertonic solution containing 1.4% NaCl and 2.5 mM EDTA, collected after gentle centrifugation and resuspended in PBS (phosphate buffered saline).

The tumour cell line DHD/K12 was obtained from a transplantable colon carcinoma induced by 1,2 dimethylhydrazine in BDIX rats (Martin *et al.*, 1983). Two sublines, named respectively DHD/K12/TR and DHD/K12/TS were selected according to their susceptibility to trypsin-mediated detachment from a plastic surface. Clones derived from these sublines and called DHD/K12/TRb and DHD/K12/TSb were used in this study. When injected into syngenic rats DHD/K12TRb cells, the most resistant to trypsin-mediated detachment, gave rise to progressive tumours in most of the animals (hence their name of PROb), whereas DHD/K12/TSb cells (named REGb), easily detached by trypsin, gave no tumours or produced tumours which regressed spontaneously in a few weeks (Martin *et al.*, 1983). These cells were grown in Ham's F10 medium supplemented with 10% foetal calf serum and 1% of antibiotic solution containing 10,000 IU $ml^{-1}$ penicillin and 10,000 UG $ml^{-1}$ streptomycin.

### *Preparation of rat plasminogen and plasmin*

Blood from 200 male adult WAG rats was withdrawn in the presence of heparin (Choay, Paris, France) by intracardiac puncture. The blood samples collected were immediately pooled with an anticoagulant solution (0.017 M citric acid, 0.087 M trisodic citrate, 0.14 M glucose, 0.02 M $NaH_2PO_4$), supplemented with 1200 TIU aprotinin and 10 $\mu$M benzamidine at pH 7.0. Plasminogen (Pg) was prepared by affinity chromatography on lysine-sepharose and further purified by anion exchange chromatography as previously described (Bertrand *et al.*, 1986). Pg was then labelled with $^{125}I$ (Amersham, Les Ulis, France) using the iodogen technique (Fraker & Speck, 1978). A specific activity of 4–8 $\mu Ci\ \mu g^{-1}$ protein (148–296 Kbq) was routinely obtained.

Non radiolabelled Pg could be activated by addition of human urokinase (Choay) (5000 IU for 10 $\mu$g of Pg) after 30 min incubation at 37°C. Ten $\mu$g radiolabelled Pg were transformed into plasmin (Pli) by the addition of 10,000 IU human urokinase. Completion of this activation was checked by PAGE followed by autoradiography. Gel electrophoresis was performed in 10% polyacrylamide gel in the presence of 0.1% sodium-dodecyl-sulphate (SDS) and 0.2 mM $\beta$ mercaptoethanol. Gels were stained by Coomassie Blue, or dried and covered with Hyper films MP (Amersham). The activation of non radiolabelled Pg appeared as complete whereas the activation of radiolabelled Pg into Pli by human urokinase was incomplete (from about 50 to 80%).

Correspondence: P. Burtin.
Received 23 July 1991; and in revised form 11 March 1992.

*Binding experiments*

Cells from PROb or REGb clones were detached from tissue culture flasks by a mixture of trypsin and EDTA, then suspended in 10 ml Ham's F10 medium supplemented with 10% foetal calf serum, centrifuged for 5 min at 1,000 r.p.m. and again suspended in the same medium. Cells were seeded in individual plastic wells (Nunclon 1/67008) ($6 \times 10^3$ per well) and grown in Ham's F 10 medium for up to 3 days without attaining confluence. Tumour cells were washed three times in PBS before a treatment with either an acidic buffer (glycine 0.05 M, NaCl 0.15 M at pH 3) 10 min at 37°C, or with a solution of 50 nM human plasmin (Kabi, Les Ulis, France), 30 min at 37°C, or with successively 10 min of acidic treatment followed by 30 min human plasmin proteolysis. Camacho *et al.* (1989) have shown that limited proteolysis of human tumour cells increased their plasmin-binding ability by increasing the number of binding sites. Comparable binding was obtained with cells treated with acidic buffer, with cells treated with human plasmin, or with cells submitted to both. Routinely, experiments were performed with human plasmin alone. Cells were then incubated for 1 h at 4°C with increasing concentrations of radiolabelled rat Pg. Specific binding was determined as the difference between total binding and binding observed in the presence of 2μM non-radiolabelled Pg. Inhibition experiments with Pli were performed in the presence of 900 KIU aprotinin (Trasylol, Sigma) in order to prevent autodegradation of Pli incubated with the tumour cells. Inhibition experiments were made using standard concentrations of $^{125}$I Pg (3 nM) with increasing amounts of cold plasminogen or plasmin, or with unrelated proteins at high concentrations such as 3μM transferrin, 4μM ovalbumin or with 10, 50 or 100 mM lysine (all from Sigma, Saint-Louis, USA). Finally the cells were washed three times with PBS and the bound ligand was solubilised with 1 N NaOH. The radioactivity was counted in a Wallac LKB γ spectrometer.

In some experiments after Pg binding at 4°C, the cells were incubated at 37°C for 20 min with a glycine buffer 5 mM pH 3, containing 0.15 M NaCl. The supernatants were studied by PAGE under reducing or non-reducing conditions followed by autoradiography.

Analogous experiments were carried out on intestinal crypt cells of control rats. Inhibition experiments were made using standard concentration of $^{125}$I Pg (1 nM) with increasing amounts of cold Pg, cold Pli or with high concentrations of unrelated proteins, such as 3μM transferrin or with 10, 50 mM lysine. Some experiments of binding were also performed with $^{125}$I human plasmin and inhibitions were made with cold human plasmin.

Scatchard plots were established from inhibition curves, using a programme that permitted calculation of the binding parameters, dissociation constant and the number of binding sites.

The enzymatic activity of cell-bound Pg or Pli was also studied. Tumour cells were seeded in 16-well Costar plates then incubated 30 min at 37°C with 100 nM human uPA, or 200 nM rat Pg alone, or with 20, 50, 100, 200 nM rat plasmin. after three washings, a synthetic ligand, H-D-valyl-L-Leucyl-L-lysine-7 amino-4 methyl coumarin (Bachem, Budendorf, Switzerland) at a concentration of 5 μg ml$^{-1}$ in PBS was added. The incubation lasted 30 min at 37°C. The fluorescence due to the release of aminomethyl-coumarin from this peptide allowed to estimate enzymatic activity. The release of fluorochrome was measured in a Jobin and Yvon spectrofluorometer (Paris, France) (λ excitation, 365 nm; λ emission, 450 nm).

*Solubilisates*

When tumour cells PROb and REGb were near confluence in 175 cm$^2$ Falcon flasks, they were washed three times with PBS, and gently scraped with 10 ml PBS containing 200 KIU aprotinin. After centrifugation 5 min at 1200 r.p.m., the pellet was incubated 20 min at room temperature in 1 ml PBS buffer containing 1% SDS + 25% glycerol and a mixture of antiproteases (100 KIU aprotinin, 0.1 mM DFP, 10 μM Pepstatin, 1 μM E64 and 5 mM EDTA, all obtained from Sigma). The solubilisates were centrifugated at 190,000 g, 1 h at 4°C. The supernatants were collected and the protein content was determined using the BCA reagent (Biorad, Richmond, CA). An amount of 500–700 μg ml$^{-1}$ was routinely obtained with both PROb and REGb cells.

*Dot blot technique and SDS-PAGE*

These solubilisates were tested for their Pg binding activity in dot blots and their contents analysed in electrophoresis followed by transfer and autoradiography as already described (Burtin & Fondanèche, 1990).

Rows of dots were incubated 1 h at 4°C with 1 nM radiolabelled rat Pg in incubation buffer. In parallel experiments radiolabelled Pg was mixed with 1 μM cold Pg or an unrelated cold protein such as 2 μM transferrin or 2 μM ovalbumin. Other samples were run in 10% polyacrylamide gels containing 0.1% SDS without previous boiling nor addition of β-mercaptoethanol. However, some samples were mixed with 0.2 M β mercaptoethanol and boiled for 5 min. Proteins were then electrically transferred to nitrocellulose sheets. These sheets were incubated 1 h at 4°C with 1 nM radiolabelled rat Pg alone or mixed with an excess of cold proteins either 1 μM of cold Pg or 2 μM of transferrin then after washings and drying covered with hyperfilms MP (Amersham). Exposure time lasted 18 h at − 80°C.

### Results

*Existence of plasminogen binding sites on PROb and REGb cells*

Radiolabelled rat Pg was weakly bound on colon cells from healthy control rats, but this fixation was not inhibited by cold Pg or Pli (Table I). Radiolabelled Pg was bound specifically by PROb and REGb cells. This binding was saturable (Figure 1) and time-dependent; the maximum of Pg binding was obtained after 1 h of incubation (for instance on

**Table I** Inhibition of radiolabelled rat Pg binding on normal colon cells

| *Cold proteins* | *% inhibition of rat Pg* binding* |
|---|---|
| Rat Pg 100 nM | 11% |
| Rat Pg 500 nM | 13% |
| Rat Pg 1 μM | 6% |
| Rat Pg 2 μM | 8% |
| Rat Pli 50 nM | 5% |
| Rat Pli 100 nM | 5% |
| Transferrin 3 μM | 11% |
| Lysine 10 mM | 42% |
| Lysine 50 mM | 65% |

Normal colon cells were incubated for 1 h at 4°C with radiolabelled plasminogen (1 nM) alone or mixed with excess of cold proteins.

**Table II** Inhibition of Pg binding on tumour cells by cold proteins

| *Cold proteins* | *PROb cells* | *REGb cells* |
|---|---|---|
| Plasminogen 200 nM | 50% | 15–20% |
| Plasminogen 1 μM | 60–70% | 50% |
| Plasminogen 2 μM | 75–90% | 70–80% |
| Plasmin 50–100 nM | 50% | 50% |
| Plasmin 1 μM | 70–85% | 70–85% |
| Ovalbumin 4 μM | 0% | 0% |
| Transferrin 3 μM | 0–10% | 0–10% |
| Lysine 50 mM | 80–90% | 80–90% |
| Lysine 100 mM | 90–98% | 90–98% |

DHD-K12 PROb or REGb cells were incubated for 1 h at 4°C with radiolabelled plasminogen (3 nM) alone or mixed with excess of cold proteins.

**Figure 1** Curves of specific binding of rat plasminogen on tumour cells PROb and REGb. Cells were incubated for 1 h at 4°C with increasing amounts of radiolabelled plasminogen either alone or with an excess of cold Pg. Specific binding was determined as the difference between total binding and binding observed in the presence of excess of cold Pg. ■ REGb; △ PROb.

**Figure 2** Curve for plasminogen-binding inhibition. PROb cells were incubated for 1 h at 4°C with radiolabelled Pg (3 nM) and various amounts of cold plasminogen (50 nM–2 μM).

PROb cells, we found a binding of 7775 d.p.m. after 15 min; 9824 d.p.m. after 30 min; 13202 d.p.m. after 1 h; 12243 d.p.m. after 2 h; 13105 d.p.m. after 3 h). A strong inhibition was observed with cold plasminogen (Figure 2) and with cold plasmin, but not with high amounts of unrelated cold proteins (Table II). A strong inhibition was also noted with lysine (50 or 100 mM), proving that Pg interacts with its receptor by the intermediate of lysine-binding sites. Basically, both sublines were able to bind radiolabelled Pg specifically but differences were observed in binding parameters.

In some experiments, the bound radiolabelled Pg was eluted from PROb or REGb cells by using a glycine buffer (pH 3). The major part (from 70 to 90%) of the bound radioactivity could be solubilised, that shows that most of the ligand was not internalised. These eluates were studied by PAGE under reducing conditions followed by autoradiography. A unique Pg band at 92 kD was observed (Figure 3).

**Figure 3** Autoradiographs after polyacrylamide gel electrophoresis of rat native ligand, rat plasminogen activated by human uPA and cell eluates. Electrophoreses were performed in 10% PAGE in presence of 0.2 mM β mercaptoethanol for 1 h 30 at 150 V. Then gels were dried and covered with Hyper film MP. Exposure lasted 2–48 h at −80°C. Lane 1, rat radiolabelled plasminogen; lane 2, radiolabelled plasmin obtained after incubation of Pg with human uPA. Lanes 3 and 4: PROb or REGb cells were incubated with 20 nM radiolabelled Pg 1 h at 4°C. After washings, cell-bound radioactivity was counted and then eluted 10 min at 37°C with glycine buffer pH 3. Lane 3, eluate of REGb cells; lane 4, eluate of PROb cells.

**Table III** Enzymatic activity of PROb cell-bound Pg or Pli

| *Product(s) added to cells* | *Fluorescence intensity Gain: 100 ×* | *Fluorescence intensity Gain: 10 ×* |
|---|---|---|
| Control: peptide alone | 0.115 | |
| Human uPA 100 nM | 0.076 | |
| Rat Pg 200 nM | 0.120 | |
| Human uPA 100 nM + rat Pg 20 nM | 0.347 | |
| Human uPA 100 nM + rat Pg 200 nM | >2 | 0.365 |
| Rat Pli 20 nM | 0.298 | |
| Rat Pli 50 nM | >2 | 0.190 |
| Rat Pli 100 nM | >2 | 0.220 |
| Rat Pli 200 nM | >2 | 0.325 |
| Rat Pli 20 nM + aprotinin | 0.108 | |
| Rat Pli 100 nM + aprotinin | 0.152 | |

Release of fluorochrome was measured in a Jobin and Yvon spectrofluorometer (λ excitation: 365 nm; λ emission: 450 nm).

The fixation of radiolabelled plasmin on DHD/K12 cells was not studied since it was not possible to activate rat radiolabelled Pg completely with high amounts of human uPA used either soluble or immobilised on CNBR-sepharose column. In fact, we obtained a mixture containing both Pg and plasmin. However experiments with cold plasmin showed that the enzyme inhibited the fixation of labelled Pg on tumour cells at lower doses than the proenzyme. An inhibition of about 50% was obtained with 50–100 nM cold plasmin (Table II), whereas 200 nM cold Pg was needed to obtain similar results with PROb cells, and 1μM cold Pg with REGb cells.

The enzymatic activity of cell-bound Pg or Pli on chromogenic substrate was estimated (Table III). Similar results were obtained with PROb or REGb cells. Fluorescence intensity was very weak when Pg alone was incubated with tumour cells. It was two to ten fold higher when cells were incubated with increasing doses of plasmin. This activity was always abolished in the presence of aprotinin. Thus, enzymatic activity of plasmin was retained after incubation with the tumour cells and the catalytic active site appeared not to be involved in the binding to cells.

Some experiments were performed on whole cells seeded on glass coverslips. After incubation with a 0.25% trypsin solution, although some cells were detached, the preparations were incubated with biotinylated Pg (usually 12 $\mu g\ ml^{-1}$ in PBS) for 30 min at 4°C as already described (Correc et al., 1992). They were reacted with fluoresceinated streptavidin. The ability of tumour cells to bind Pg was not altered since fluorescent patterns were observed on the outlines of remaining cells and on the extracellular matrix, as observed on untraited cells (data not shown).

### *Characteristics of binding sites on PROb or REGb cells*

Radioligand displacement assays by cold Pg performed on PROb cells gave the following results: 65%–80% inhibition in the presence of 1 μM cold Pg *vs* 0–10% in the presence of PBS or high amounts of cold ovalbumin or human transferrin. Pg binding was inhibited 50% by 200 nM cold Pg.

Using the data from ten inhibition experiments, the dissociation constant of Pg was calculated according to Scatchard's method. The Kd was about $1.0 \times 10^{-7}$ M. The number of binding sites was $0.9 \times 10^6$ per cell (range from 0.6 to $1.2 \times 10^6$ per cell).

Analogous experiments were performed with REGb cells (Table II). Pg binding was inhibited 50% by 1 μM cold Pg. Using the data from ten inhibition experiments, the dissociation constant was about $5 \times 10^{-7}$ M. The number of binding sites was about $3.6 \times 10^6$ per cell (range from 1.2 to $6.10^6$ by cell). The Figure 4 shows the comparison of the two Scatchard plots obtained respectively with PROb and REGb cells.

**Figure 4** Comparison of Scatchard plots of plasminogen-binding inhibition obtained with PROb and REGb cells. ◀ PROb; ■ REGb.

### *Dot blots*

Ability of solubilisates to bind radiolabelled rat Pg specifically was determined by the counting of dot blots. Five replicates were made for each experiment. Using the data from six different experiments, it appeared that the fixation of radiolabelled Pg was inhibited by 50–70% on PROb and by 60–80% on REGb solubilisates with 1 μM cold rat Pg. This inhibition was dose-dependent since for instance with REGb solubilisates, it was only 50% with 700 nM cold Pg and 40% with 500 nM cold Pg. The fixation of rat Pg was not inhibited with 2 μM transferrin or ovalbumin. However, in some experiments transferrin showed minor inhibition which was subtracted from that given by cold rat Pg in order to calculate specific inhibition.

### *PAGE analysis of SDS solubilisates*

Autoradiograms obtained after incubation of nitrocellulose sheets with radiolabelled rat Pg showed one major band in the 50–60 kD area (Figure 5). In some cases two other fainter bands were also visible in the 30 kD area. After incubation with radiolabelled Pg mixed with 1 μM cold Pg (1000 fold excess of cold Pg) no band was visible. No inhibition was noted if radiolabelled Pg was mixed with 2 μM (2000 fold excess) cold transferrin (Figure 5). Identical patterns were always observed on both PROb and REGb solubilisates. The patterns were the same if SDS solubilisates were boiled or treated with β-mercaptoethanol or both, in particular the band situated in the 50–60 kD area was still visible.

### Discussion

Our results demonstrate that the two PROb and REGb cancer cell lines can bind rat Pg in a time-dependent, specific and saturable manner as do several human tumour lines with human Pg (Burtin & Fondanèche, 1988; Corre et al., 1990). This study has been impeded by technical difficulties since rat uPA is not available commercially. When we used human

**Figure 5** Characterisation of plasminogen receptor obtained from solubilisates of PROb cells. After electrophoresis on 10% PAGE, 1 h 30 at 150 V, all samples were transferred on nitrocellulose sheets. These sheets were incubated 1 h at 4°C with different proteins, washed, dried, and covered with Hyper film MP. Exposure lasted 18 h at − 80°. Lane 1, incubation of sheet with 1 nM radiolabelled rat Pg. Lane 2, incubation with a mixture of 1 nM radiolabelled Pg and 1 μM cold Pg. Lane 3, incubation with a mixture of 1 nM radiolabelled Pg and 2 μM human transferrin.

uPA, the activation of rat Pg was complete except when this Pg was radiolabelled with $^{125}$I. Thus, it has not been possible to study the binding of radiolabelled rat plasmin. However, inhibition experiments seem to show that the same molecules bind Pg or Pli with an higher affinity for Pli since the 50% inhibition concentration of Pg binding is significantly smaller for Pli than for Pg. That agrees with the results obtained on human carcinoma cells (Burtin & Fondanèche, 1988; Correc *et al.*, 1990). It was shown that the same receptors bound human Pli and Pg but the amount needed to inhibit the radiolabelled human Pg binding by 50% was significantly lower for Pli than for Pg. Note that these human receptors linked human Pg with a lower affinity than those detected for rat Pg on PROb or REGb cells (50 fold lower for SW1116 cells and 10 fold for MCF7 cells).

Another piece of evidence shows that Pli is bound by PROb and REGb cells: the cells incubated with cold rat Pli acquired protease activity. That indicates also that the catalytic active site of Pli is not required for its binding to cancer cells. Furthermore, the rat Pli used to inhibit the binding of radiolabelled Pg was always supplemented with aprotinin which blocks the Pli active site, but does not prevent its competitive effect. Identical results have been previously obtained with human carcinoma cells (Burtin & Fondanèche, 1988; Correc *et al.*, 1990) which suggested that the Pli binding on these rat carcinoma cells was also due to a specific receptor and not to protease-nexin like molecules.

It was not possible to study the receptor for urokinase on PROb and REGb cells, since rat uPA is not available, and it was reported that the receptor for uPA is species-specific (Appella *et al.*, 1987; Estreicher *et al.*, 1989). However on human carcinoma cell line SW 1116, Burtin & Fondanèche (1990) have shown that receptors for Pli and for uPA are distinct proteins, since a high amount of urokinase does not inhibit the specific binding of plasmin. As we have shown a strong interspecific reactivity between the receptors for Pli and for Pg on human and rat carcinoma cells (Durliat *et al.*, 1991), it is likely that the two systems are homologous and that rat Pg receptor is distinct from rat uPA receptor.

It is clear that Pg binding sites are surface molecules. Two types of experiments support this conclusion. First, after binding of radiolabelled Pg, acid elution leads to recovery of 70–90% of the bound ligand. Second, fluorescence experiments showed the staining of the surface of whole tumour cells. Note also that on several human tumour cells (SW 1116 and MCF7-MF), Correc *et al.* (1992) have evidenced images with green fluorescence clearly visible as grains or contours at the surface of these tumour cells. Moreover, Pg binding sites are membrane molecules since dissolved by detergent, but not by hypertonic saline buffer.

Pg binding sites of high capacity, of similar affinities and with common recognition specificities are also reported to be widely distributed on many human blood cells with Kd values of 0.9–1.4 $\mu$M (Miles & Plow, 1987), and on both promyeloid leukaemic U 937 and diploid foetal lung GM 1380 fibroblast cell lines (Plow *et al.*, 1986).

The functional role of these rat Pg binding sites has not been unambiguously determined on tumour cells. It is likely that these binding sites serve to focus high amounts of Pg on the surface of certain cells which could also bind uPA. Activation of bound Pg by uPA could proceed in a very effective way, thus generating abundant proteolytic activity for the degradation of pericellular substrates, facilitating the migration of tumour cells. Considering the great difference in malignancy of PROb and REGb cells, we were interested in determining whether the binding sites for Pg and/or for Pli exhibited identical or different characteristics in the two cell sublines. In fact, a higher affinity ( $\times$ 5) for Pg has been observed on PROb than on REGb cells, but with a parallel decrease in the number of binding sites. It is possible that low and high affinity Pg receptors are different forms of the same molecule as postulated for the receptors of uPA (Estreicher *et al.*, 1989). Thus no significant difference in the specific binding of rat Pg on these two cell lines was detected which might be related to their degree of malignancy.

Analyses of solubilisates obtained from both PROb and REGb cells show that these samples contain specific binding sites for radiolabelled rat Pg. In electrophoresis, these receptors exhibited the same mobility than those detected for Pli and Pg on human epithelial tumour cell lines (Burtin & Fondanèche, 1990; Correc *et al.*, 1990).

We think that the cell surface Pg binding protein detected in experiments dealing with whole cells corresponds to that found in cell solubilisates. We found only one Pg binding band in Western blots performed with cell solubilisates. This means that this band is similar to the cell surface molecule, unless this latter molecule was denatured during solubilisation process. Other evidence is derived from experiments performed on human MCF7 tumour cells (P. Correc, unpublished results). The Pg receptor was studied by cross-linking technique and solubilisation with SDS. For the complex between Pg and receptor, a molecular weight of 140–150 kDa was obtained after polyacrylamide gel electrophoresis and autoradiography. This indicates a molecular weight of 50–60 kDa for the receptor molecule. As the rat and human receptors are very similar (Durliat *et al.*, 1991), we believe that the rat Pg receptor has indeed the same molecular weight, similar to that obtained on blotting experiments.

To conclude, this study is the first characterisation of specific Pg binding sites on rat carcinoma cell lines.

### References

APPELLA, E., ROBINSON, E.A., ULLRICK, S.J., STOPELLI, M.P., CORTI, A., CASSANI, G. & BLASI, F. (1987). The receptor-binding sequence of urokinase. *J. Biol. Chem.*, **262**, 4437.

BERTRAND, O., COCHET, S., KROVIARSKI, Y., TRUSKOLASKI, A. & BOIVIN, P. (1986). A low cost modular apparatus of large applicability for automation of chromatographies. The example of automated purification of plasminogen and histidine rich glycoprotein from the same plasma sample. *Protein purification technologies*. 2nd Sym. Europ., Nancy 29 Sept.–2 Oct., p. 205.

BURTIN, P., CHAVANEL, G. & ANDRE, J. (1985). The plasmin system in human colonic tumors; an immunofluorescence study. *Int. J. Cancer*, **35**, 307.

BURTIN, P., CHAVANEL, G., ANDRE-BOUGARAN, J.& GENTILE, A. (1987). The plasmin system in human adenocarcinomas and their metastases. A comparative immunofluorescence study. *Int. J. Cancer*, **39**, 170.

BURTIN, P. & FONDANECHE, M.C. (1988). Receptor for plasmin on human carcinoma cells. *J. Natl Cancer Inst.*, **80**, 762.

BURTIN, P. & FONDANECHE, M.C. (1990). Solubilization of the plasmin receptor from human carcinoma cells. *Biochem. Biophys. Res. Commun.*, **170**, 748.

CAMACHO, M., FONDANECHE, M.C. & BURTIN, P. (1989). Limited proteolysis of tumor cells increases their plasmin-binding ability. *FEBS Lett.*, **245**, 21.

CORREC, P., FONDANECHE, M.C., BRACKE, M. & BURTIN, P. (1990). The presence of plasmin receptors on three mammary carcinoma MCF-7 sublines. *Int. J. Cancer*, **46**, 745.

CORREC, P., ZHANG, S., KOMANO, O., LAURENT, M. & BURTIN, P. (1992). Visualization of the plasmin receptor on carcinoma cells. *Int. J. Cancer*, **50**, 767.

DANO, K., ANDREASEN, P.A., GRONDAHL-HANSEN, J., KRISTENSEN, P., NIELSEN, L.S. & SHRIVER, L. (1985). Plasminogen activators, tissue degradation and cancer. *Adv. Cancer Res.*, **44**, 140.

DURLIAT, M., KOMANO, O., CORREC, P. & BURTIN, P. (1991). A receptor for plasminogen and plasmin on human and rat carcinoma cells. Evidence for strong interspecific reactivity. *Thromb. Res.*, **64**, 637.

ESTREICHER, A., WOHLWEND, A., BELIN, D., SCHLEUNING, W.D. & VASSALI, J.D. (1988). Characterization of the cellular binding site for the urokinase-type plasminogen activator. *J. Biol. Chem.*, **264**, 1180.

FRAKER, P.J. & SPECK, J.C. (1978). Protein and cell membrane iodinations with a sparingly soluble chloramide, 1,3,4,6-tetrachloro-3a,6a- diphenylglycoluril. *Biochem. Biophys. Res. Commun.*, **80**, 849.

MARTIN, F., CAIGNARD, A., JEANNIN, J.F., LECLERC, A. & MARTIN, M. (1983). Selection by trypsin of two sublines of rat colon cancer cells forming progressive or regressive tumors. *Int. J. Cancer*, **32**, 623.

MILES, L.A. & PLOW, E.F. (1987). Receptor mediated binding of the fibrinolytic components, plasminogen and urokinase, to peripheral blood cells. *Thromb. Haemost.*, **58**, 936.

MULLINS, D.E. & ROHRLICH, S.T. (1983). The role of proteinases in cellular invasiveness. *Biochim. Biophys. Acta*, **695**, 177.

OSSOWSKI, L. (1988). Plasminogen activator dependent pathways in the dissemination of human tumor cells in the chick embryo. *Cell*, **52**, 321.

PLOW, E.F., FREANEY, D.E., PLESCIA, J. & MILES, L.A. (1986). The plasminogen system and cell surfaces: evidence for plasminogen and urokinase receptors on the same cell type. *J. Cell. Biol.*, **103**, 2411.

SAKSELA, O. & RIFKIN, D.B. (1988). Cell-associated plasminogen activation: regulation and physiological functions. *Ann. Rev. Cell. Biol.*, **4**, 93.

SALO, T., LIOTTA, L.A., KESKI-OJA, J., TURPEENNIEMI-HUJANEN, T. & TRYGGVASON, K. (1982). Secretion of basement membrane collagen degrading enzyme and plasminogen activator by transformed cells – Role in metastasis. *Int. J. Cancer*, **30**, 669.